# Mitochondrial sequence based characterization and morphometric assessment of Diara buffalo population

**DOI:** 10.5713/ab.21.0265

**Published:** 2022-01-05

**Authors:** Karan Veer Singh, Hitesh Purohit, Ramesh Kumar Singh

**Affiliations:** 1 National Bureau of Animal Genetic Resources, Karnal, Haryana 132001 India; 2 Department of Animal Genetics and Breeding, Bihar Veterinary College, Patna, Bihar 800014, India

**Keywords:** Buffalo, Genetic Diversity, Molecular Markers, Morphometric

## Abstract

**Objective:**

The present study is aimed at phenotypic characterization and mitochondrial d-loop analysis of indigenous “Diara” buffalo population, which are mostly confined to the villages on the South and North Gangetic marshy plains in the Bihar state of India. These buffaloes are well adapted and are best suited for ploughing and puddling the wet fields meant for paddy cultivation.

**Methods:**

Biometric data on 172 buffaloes were collected using a standard flexible tape measure. Animals are medium in size; the typical morphometric features are long head with a broad forehead and moderately long and erect ears. Genomic DNA was isolated from unrelated animals. The mtDNA d-loop 358-bp sequence data was generated and compared with 338 sequences belonging to riverine and swamp buffaloes.

**Results:**

Based on the mitochondrial d-loop analysis the Diara buffaloes were grouped along with the haplotypes reported for riverine buffalo. Sequence analysis revealed the presence of 7 mitochondrial D loop haplotypes with haplotype diversity of 0.9643. Five of the haplotypes were shared with established swamp breeds and with Buffalo population of Orissa in India.

**Conclusion:**

Morphometric analyses clearly shows distinguishing features like long and broad forehead which may be useful in identification. The germplasm of Diara buffalo is much adapted to the marshy banks of river Ganga and its tributaries. It constitutes a valuable genetic resource which needs to be conserved on priority basis.

## INTRODUCTION

The buffalo plays a very important role in the South Asian region economically and culturally. This region has a great biodiversity of buffalo, constituting 56% of the world buffalo population, which includes the well-known Murrah and Nili-Ravi breeds [1,2]. India is home to the high milk producing breeds of buffalo of the world. There are 19 well recognized buffalo breeds which constitute about 30% of the total buffalo population in the country [3]. However, 70% of the buffalo population in the country are still to be characterised and are known as non-descript. They possess the highest potential for production with a promising gene pool, which is still not fully utilised.

Mitochondrial genome is inherited through maternal lineage only, the DNA displacement loop of the mitochondrial genome (mtDNA D-loop) has been a prime target to carry out molecular evolutionary studies [4]. The higher mutation rate of mtDNA as compared to that of single copy nuclear genes makes mtDNA prime target for phylogenetic studies. It has provided significant insights into the domestication and past migration history of various livestock species.

Genetic studies using mitochondrial DNA (mtDNA) and the present-day distribution of buffalo breeds around the world have shown that swamp and river buffalo have been domesticated independently [1,5]. Based upon mitochondrial control region and cytochrome b sequence analysis it has been proposed that these two domestic types would have been derived independently from their respective wild ancestors that would have differed from each other at least at the level of subspecies [1]. Nagarajan et al [6] have analyzed 492-bp mtDNA control region sequences of riverine buffaloes sampled from India, Pakistan, Egypt, and Iran along with the available swamp sequences and provided genetic evidence that river buffalo were domesticated in Indian subcontinent and subsequently have reached Mesopotamia in ancient times from India. Earlier studies based on the *TLR8* gene showed Assamese buffalo to be closer to the riverine buffaloes of eastern region like Chilika, Kalahandi, and Diara, as compared to buffalo population of Northern, Western, or Southern India [7].

River Ganga enters the state of Bihar from Chausa and divides state into two parts. Many of its tributaries Gandak, Bagmati, Kosi, Kali, Sone, Karmanasa, and Punpun enter river Ganga on its onward flow. The drainage pattern of Bihar is both trellis and dendritic. The villages on the South and North banks are densely populated with clusters of buffalo [8]. South and North Gangetic plains of river Ganga gets annual floods and is submerged under water. The waterlogged marshy habitation is well suited for these buffaloes which are well adapted to Taal and Diara areas of the river Ganges and its tributaries Sone and Gandak. Based on physical attributes, the farmers usually call these buffalo Deshila or “Diara buffalo”, or Punjabiya (graded Murrah). Diara buffalo are good milkers producing 7.8 litre average per day milk. The population remains so far largely untouched, management and breed improvement programmes could be undertaken to further improve the genetic potential of these buffaloes [8]. Characterisation of non-descript livestock and poultry populations are a mandated activity of ICAR-NBAGR. Hence the present study was undertaken to characterise this non-descript indigenous population based on morphometric and Mitochondrial d-loop analysis.

## MATERIALS AND METHODS

### Ethics statement

The study was approved by the Animal Ethics committee 2019-20/IRC-2.11, ICAR-National Bureau of Animal Genetic Resources, Karnal. All methods were carried out in accordance with guidelines and regulations of the concerned committee.

### Morphometric data collection

A survey was conducted in villages of Patna, Ara, Chhapra, and Buxar districts situated on the banks of river Ganga in the Bihar state to collect and record information on various management practices of Diara buffalo. The information and management practices were collected by visiting 120 farmers of the 16 villages in the breeding tract based on standard procedure developed by ICAR-NBAGR for breed survey and registration.

Reproduction traits of Diara buffaloes were estimated based on the information provided by the farmers who rear these buffaloes. Performance traits like age at first calving, age at first service, daily milk yield, calving interval, lactation yield, peak yield, lactation length, dry period, service period and calving interval were recorded. Standard body measurements and physical characteristics were recorded from 132 animals of different age and sex, including calves. The body measurements data was analysed for descriptive statistics using MS-Excel [9]. Physical characterization of adult buffaloes and phenotypic correlations between different body biometric traits were estimated using partial correlations, using the SPSS (2001) [10].

### Sample collection and genomic DNA isolation

Blood samples from the jugular vein of unrelated buffalo of both sexes were collectedfor mitochondrial DNA D-Loop analysis from various villages in the Diara buffaloes’ breeding region. DNA was isolated following standard protocol of SDS-Proteinase-K described by Sambrook and Russel [11]. The primers used to amplify mitochondrial D-loop segment were designed from buffalo complete mitochondrial sequences available in the GenBank database (Accession No. AF547270).

The primer set was F: 5′ AGTCCAAGCATCCCCCAAAATAAA 3′; R: 5′ CGGCCAGCATAATCGAAA 3′. The polymerase chain reaction (PCR) cycling conditions used were initial denaturation at 95°C for 3 min followed by 32 cycles of 94°C for 30 s; 58°C for 30 s; 72°C for 1 min with final extension at 72°C for 10 min. The amplified products were analysed on 1% TAE-agarose gel. After purification of PCR products, sequencing was performed using BigDye Terminator 3.1 Cycle Sequencing Kit (Applied Biosystem, Foster City, CA, USA) on an ABI 3130xl Automated DNA sequencer.

### Sequencing and sequence analysis

Multiple sequence alignment of the edited nucleotide sequences was performed using MEGALIGN programme of Lasergene (DNASTAR Inc., Madison, WI, USA) [12]. Nucleotide sequence analysis of mtDNA D-loop haplotype sequences of Diara buffaloes was carried out by comparing with other reported Indian riverine populations (Assamese, Chilika, Kalahandi, Paralakhmundi, Marathwada, Murrah, South Kanara, Toda, Chhattisgarh, Dharwadi, Sambalpuri, and Manda) as well as a swamp population Meghalaya, Manipuri, Laos, Egypt, and Bangladeshi buffaloes. Mitochondrial D-loop haplotype sequence data for these populations were retrieved from the GenBank database, Chinese buffaloes, Carabao and Mediterranean buffaloes, while *Bostaurus* and *Bosindicus* were used as out-group [13–16]. The evolutionary history was inferred using the Neighbour-Joining method [17]. Phylogenetic analyses were conducted in MEGA7 [18]. DNASP version 5.0 [19] was employed to analyze various genetic diversity parameters. NETWORK version 5.0 was utilized to draw the reduced median network [20].

## RESULTS AND DISCUSSION

Diara buffaloes are smaller than the heavy-sized breeds like Murrah, Jaffarabadi, and Nili-Ravi with a height at withers of (138.12±4.5cm). The body hair coat colour varies from black to grey. The head is long with a broad forehead. Ears are moderately long and erect. The neck is moderately long. Horns are medium in size, flat, corrugated and curved, projecting backward, sideward, and upward reaching up to half of the neck. The tail is long, thin, and flexible ending in a black or black and white switch. Some animals showed white hairs on the forehead as well as on the forelimbs or in the pastern regions of hind limbs.

The Diara buffaloes are reared by farmers along the Ganga River. The breeding region of Diara buffaloes is almost linear along both sides of banks of river Ganges and its tributaries in Bihar. The region is almost marshy most of the year and dry enough for cultivation only during dry-weather conditions. Adult male animals are used for agricultural work in diara region, they are better suited for ploughing and puddling the wet fields meant for paddy cultivation, whereas females are used for milking. The herd size of Diara buffaloes is very small with an average being 3.1. The herd structure included almost nil adult males, 2.0 adult females, 0.2 male calves and 0.8 female calves. The black coloured animals are preferred over grey coloured by local farmers. Diara buffalo population has been influenced by continuous inter-se breeding within a population over a long period of time and inter-breeding with Murrah [21].

### Body measurements

The mean and standard error of different body measurements in different age groups were recorded (Table 1). For adult male Diara buffalo of age above the three year the height at withers, was (138.12±4.5 cm), body length (130.26±5.1 cm), heart (chest) girth (208±2.5 cm), and for adult female animal it was found to be 129.77±2.24, 117.77±3.54, 192.31±5.67, respectively. There was no significant difference between male and female Diara buffalo animals based on morphometric traits. Chandran et al [8] studied morphometric and body weight traits of Diara buffaloes of different age groups.

The height at withers, body length and heart (chest) girth was 94.25±5.00 cm, 76.37±6.89 cm, and 117±10.12 cm in the 1 year age group calves. Moioli and Borghese [22] reported the height at withers in Murrah (142 cm) and Nagpuri (140 cm) in adult male buffaloes and 133 cm and 130 cm in adult female buffaloes, respectively, which are similar to the height at withers in Diara buffalo animals found in the study. Shankar and Mandal [23] have also reported the adult body weight of (461.789±3.32 kg) in Diara buffaloes.

### Housing

Diara buffalo animals are housed close to the human dwellings. In most cases, closed housing is provided (85%) and the animals and humans are housed in different parts of the same building. Most of the constructions are permanent (85%) with thatched roofs covered with paddy straw, asbestos sheets, or tiled roofs. Floors are generally uneven brick layered without proper drainage facilities. The practice of allowing the animals to wallow in the nearby water sources is prevalent (90.5%). Traditional practice of taking animals for grazing in the marshy area of river Ganga and its tributaries is most prevalent, in the villages situated near the banks of water sources. In the evening, animals are brought in the closed house, milking is done in the evening as well as in the morning.

Stall feeding is also practiced by farmers having 2 to 3 animals, paddy straw, dry mixed grasses and green grasses are the main sources of roughage. Wheat bran, linseed oil cake, mustard oil cake and rice bran are given as concentrates. About two third of the farmers provide concentrates to the milking animals; 0.5 to 2 kg of concentrate is usually given to the lactating animals at the time of milking.

### Breeding

Artificial insemination is primarily used for breeding and is now getting priority due to unavailability of breeding bulls and other practical difficulties faced by farmers. Semen of Diara buffaloes is not available and the farmers have to opt for Murrah semen, as a result, the proportion of graded Diara buffaloes (crossbred) and non-descript animals are more common in the urban areas.

### Production performance

The udder is moderately developed with teats of medium size and squarely placed between the hind legs. Diara buffaloes are moderate milk producers and daily yield is 4 to 9 kg. The average daily milk yield was 4.9±0.4 kg as reported by the farmers. The length of lactation varied from 210 to 340 days per year with an average of 301.6±10.3 days per year. The lactation milk yield varied from 1,008.4±95.7 to 1,635.6 ±112 kg with a mean of 1,450.87±28.7 kg. Diara buffaloes have relatively long productive life spans, animals with more than five calvings were commonly found in the villages. Age at first calving and calving interval was estimated to be 46.27 ±0.63 months and 14.4±0.13 months, respectively. The dry period, average age at first service and service period was estimated to be 89.87±4.25 days, 34.86±0.78 months and 131.31 ±3.06 days, respectively. The different traits estimated above agree with Chandran et al [8].

### Analysis of mitochondrial DNA D-loop sequence variation

**Figure 1:**
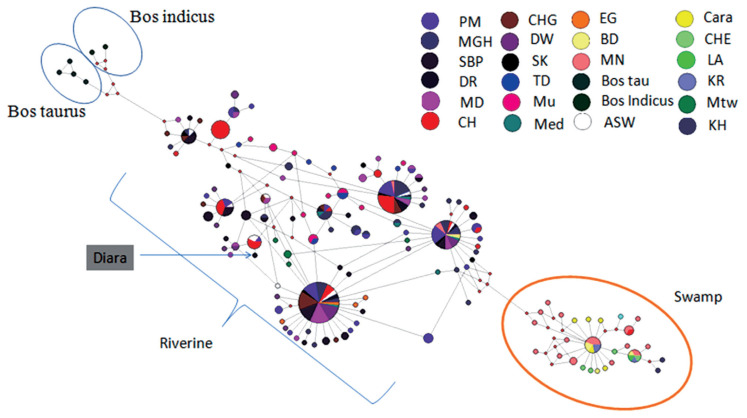
Network analysis using NETWORK 5.0, displaying median joining network based on, Mitochondrial D-loop haplotype of Diara buffalo with riverine buffalo, each haplotype is represented by a circle and the area of the circle is proportional to its frequency. Samples from different regions are mentioned in different colours. PM, Parakmundi; MGH, Meghalaya; SBP, Sambalpuri; DR, Diara; MD, Manda; CH, Chilika, CHG, Chatisgahri; DW, Dharwadi; SK, South Kanara; TD, Toda; Mu, Murrah; Med, Mediterranean; EG, Egypt; BD, Bangladesh; MN, Manipur; ASW, Assam; Cara, Carabao; CHE, Chinese; LA, Laos; Mtw, Marathwada; KH, Kalahandi.

Polymerase chain reaction successfully amplified the mitochondrial D-loop region of the buffalo samples. The two sequencing fragments were aligned and combined to produce a 358 bp mitochondrial D-loop DNA. The mtDNA D-loop sequences analysis of the sampled animals revealed seven haplotypes. Among the haplotypes found in Diara buffaloes, two haplotypes were distinct and one is shared only with Sambhalpuri buffalo while four haplotypes were found to be shared with Assam, Manipur and Meghalaya population from north-east India and also with Chilika, Kalahandi, Paralakhemundi, and Sambhalpuri breeds of Orissa, which has the character of wallowing in water and show similar habitat, these four haplotypes were also shared with Bangladesh, Mediterranean, and Egypt population which are established swamp population. The Diara buffaloes were grouped along with the haplotypes reported for riverine buffalo (Figure 1).

Mitochondrial D-loop sequence data of Diara buffaloes from Bihar were compared with other reported Indian riverine, Chinese, and Bangladeshi buffalo populations belonging to 23 different breeds (N = 342). Sequence analysis revealed the presence of 208 haplotypes, with a haplotype diversity of 0.9529±0.0045 and a nucleotide diversity of 0.03175±0.00126. The haplotype diversity ranged from 1 in South Kanara, Laos, and Egypt buffalo population to 0.7974 in Chhattisgarhi Indian population (Table 2). The genetic diversity across the different breeds followed the descending order pattern as per diversity richness, being highest in the northeast populations and lowest in central Indian riverine buffaloes. The Diara buffaloes showed highest values for nucleotide and haplotype diversity then Murrah buffaloes.

## CONCLUSION

Diara buffalo population could be an offshoot from cross between Murrah and local buffaloes and it might have evolved due to continuous inter-se breeding within population over a long period of time. Morphometric and morphology analyses clearly show distinguishing features like fairly long and broad forehead which may be useful in identification. The animals are maintained in a low input small scale farming system and are vital for livelihood support to the farmers living on the banks of yearly flooding Ganga River. We have reported genetic diversity of the Diara buffalo population based on mitochondrial markers. The Diara population is under continuous threat in their breeding territory due to artificial insemination with the elite breeds. Intervention is needed to develop exclusive semen bank of genetically superior Diara buffalo bulls. Much data is still lacking for this indigenous population. Present study analysis may be used further for the Diara buffalo characterization for breed registration.

## Figures and Tables

**Table 1:** Mean±standard error of morphometric traits of Diara buffaloes in the breeding tract

Parameters (in cm)	Young calves (<2 wk)	Calves (6–12 mo)	Young stock (1 to 3 yr)	Adults	
Animal No	10	12	12	Male (8)	Female (90)
Height at wither	80±2	94.25±5.00	111.8±7.48	138.12±4.5	129.77±2.24
Body length horizontal	52.5±3.5	76.37±6.89	89.2±7.51	130.26±5.1	117.77±3.54
Body length oblique	57.5±4.5	87±5.69	101.20±9.24	141.25 ±5.2	129±3.50
Heart girth	80.5±2.5	117±10.12	150.8±15.24	208±2.5	192.31±5.67
Paunch girth	81.5±2.5	137±10.78	167.2±14.48	228±2.89	219.09±6.12
Leg	53.5±2.5	60±1.81	69±4.33	81.21±1.9	76.09±1.61
Neck	15±1	30.87±1.75	37.8±2.41	53.26±3.9	44.63±1.43
Neck circumstance	45±1	62.37±7.02	72.6±9.9	95.16±5.9	87±2.11
Face length	21±4	31.87±1.65	36.8±2.2	44.25±1.34	40.22±1.96
Face width	9.5±0.5	13.87±0.58	17.4±0.97	23.89±3.84	19.6±0.69
Ear length	15.5±2.5	22±0.96	25±0.89	27.20±1.29	26.09±0.95
Horn length	-	8.14±2.56	21.2±3.24	34.3±1.98	32.1±2.08
Horn circumstance	-	11.28±1.10	18±1.37	21.16±1.01	18.47±1.12
Distance between horns	-	18.71±1.59	23.6±2.65	29.4±1.2	26.80±0.92
Hip bone	16.5±1.5	22.37±1.06	32.4±3.52	55.30 ±3.5	47.14±2.17
Hip height	75.19±3.83	106±16.5	112±16	135.46±2.75	126.41±1.95
Pin bone	8.5±0.5	12.25±1.57	21.2±4.95	26.9±1.95	25.4±1.61
Distance between hip and pin bone	22±2	26.37±1.96	31.2±2.47	40.23±2.3	37.3±1.54
Tail length	37.5±4.5	60±4.73	68±5.54	88.45±3.9	85.05±2.59
Tail length up to switch	43.5±4.5	70.25±5.75	77.8±5.01	97.79±3.5	95.65±3.33

**Table 2:** Mitochondrial D-loop haplotype diversity across buffalo populations/breeds

Sr. No.	Population	N	H	Hd	Pi
1	Assamese	10	8	0.95556	0.01441
2	Chilika	41	13	0.8659	0.02025
3	Kalahandi	31	15	0.9161	0.01833
4	Paralakhemundi	43	21	0.9413	0.01537
5	Manipuri	24	17	0.9638	0.03624
6	Marathwada	5	4	0.9000	0.00412
7	Murrah	12	9	0.9545	0.01114
8	South Kanara	7	7	1.0	0.01870
9	Toda	7	6	0.9524	0.01249
10	Chhattisgarh	18	9	0.7974	0.01719
11	Dharwadi	20	11	0.8684	0.01327
12	Sambalpuri	40	20	0.9474	0.01463
13	Manda	24	14	0.8877	0.01667
14	Egypt	5	5	1.0	0.00104
15	Chinese	6	5	0.9333	0.00672
16	Bangladesh	5	4	0.9000	0.03152
17	Mediterranean	6	6	1.0000	0.01430
18	Laos	5	5	1.0	0.00517
19	Carabao	6	6	1.0000	0.00861
20	Bosindicus	2	2	1.0000	0.01781
21	Bostaurus	4	4	1.0000	0.00339
22	Diara	8	7	0.9643	0.01892
23	Meghalaya	13	10	0.9487	0.02842
Total	-	342	208	0.9529	0.03175

N, number of animals; H, number of haplotypes; Hd, haplotype diversity; Pi, nucleotide diversity.
